# Variants in the ciliopathy gene SCLT1 are associated with non-syndromic retinal degeneration

**DOI:** 10.21203/rs.3.rs-6507107/v1

**Published:** 2025-05-19

**Authors:** Riccardo Sangermano, Kaoru Fujinami, Suk Ho Byeon, Emily M. Place, Julien Navarro, Christel Condroyer, Stephanie DiTroia, Christina Zeitz, Isabelle Audo, Kinga M. Bujakowska, Jinu Han

## Abstract

Inherited retinal degenerations (IRDs) are a group of clinically and genetically heterogeneous blinding disorders. In this retrospective study, we describe five families with non-syndromic IRD in which affected probands carried rare bi-allelic variants in *SCLT1* , a gene previously associated with multiple recessive ciliopathies. Seven of the eight variants identified were novel, and four of them affected splicing, including the known missense p.(Lys544Arg), detected heterozygously in three East Asian probands, and the novel, hypomorphic, deep-intronic variant c.290 + 2732A > G, leading to the inclusion of a 45-bp cryptic exon containing a premature termination codon. Analysis of the genomic data also revealed a large in-frame tandem duplication spanning exon 3–10, which was subsequently validated. Although no clear correlation was found between the severity of the *SCLT1-* associated phenotypes and the identified causal variants, this report expands the current knowledge of *SCLT1* -associated disease by enriching its mutational landscape and supports its association with non-syndromic retinal degeneration.

### INTRODUCTION

Inherited retinal degenerations (IRDs) are a group of genetically and clinically heterogeneous Mendelian disorders characterized by a progressive loss of retinal rod and cone photoreceptors, specialized neuronal cells initiating vision. The incidence of IRDs has been estimated to be around 1 in 2000 individuals, with more than two million people affected worldwide[1]. IRDs can manifest as an isolated phenotype where only the retina is affected (i.e., non-syndromic IRDs), such as in rod-cone dystrophy (RCD), or as a syndromic disease, where retinal degeneration is one of many signs of a multiorgan clinical manifestation[2]. Syndromic IRDs often arise from defects in the primary cilium, a microtubule-based sensory organelle, that projects from the plasma membrane of nearly every human cell, including the photoreceptors[3]. Absence or functional alteration of any of the primary cilium components leads to a spectrum of severe clinical manifestations, referred to as ciliopathies, affecting millions of people worldwide[4].

The Sodium Channel and Clathrin Linker 1 *(SCLT1)* gene (OMIM# 611399), initially known as clathrin-associated protein-1A (*CAP-1A*) is a ciliary gene encoding a multidomain protein. SCLT1 was first discovered as a specific interactor of the voltage-gated sodium channel $Na_v1.8$ in nociceptive neurons, where it acts as a linker between clathrin and the sodium channel in multiprotein complexes, suggesting a role as endocytic adaptor protein regulating $Na_v1.8$ density at the cell surface[5]. SCLT1 was later shown to be involved in ciliogenesis, as one of the core distal appendage (DAP) components of centrioles, a group of five proteins (CEP83, CEP89, SCLT1, CEP164, and FBF1) needed to anchor the primary cilium to the plasma membrane and promote cilia initiation[6]. In particular, SCLT1 is a key DAP member, as it is required for the recruitment of CEP164 and FBF1 at the mature centriole, also known as basal body[6].

Bi-allelic pathogenic variants in the *SCLT1* gene cause several recessive ciliopathies, such as orofaciodigital syndrome type-IX (OFD-IX; OMIM# 258865), characterized by developmental defects of the oral cavity (e.g., cleft palate), facial features, digital abnormalities, and retinal defects[7,8], Bardet-Biedl syndrome (BBS; OMIM PS209900), characterized by RCD, truncal obesity, cognitive impairment, hypogonadism in men, digital and renal abnormalities[9], and Senior-Løken syndrome (SLS; OMIM PS266900), hallmarks of which are nephronophthisis and early-onset severe retinal dystrophy (EOSRD)[10,11].

Many ciliopathy cases harboring pathogenic *SCLT1* variants have been described to date[12–21]. However, in 2016, DeCastro Miro' and colleagues described two siblings with early-onset RCD in one Argentinian family carrying compound heterozygous variants in *SCLT1*[22], and very recently a third compound heterozygous non-syndromic IRD patient from a large mutational screen of 1000 affected cases[23]. However, a conclusive association of *SCLT1* mutations with non-syndromic IRD has never been reached due to the small number of non-syndromic cases. In this study, we describe four new families with non-syndromic retinal degeneration and one additional case with a milder form of OFD-IX that confirm the phenotypic expansion for bi-allelic variants in *SCLT1.* We also report seven novel variants in this gene, three of which are likely pathogenic.

### RESULTS

#### Rare SCLT1 variants associated with non-syndromic IRD

By analyzing data from either targeted next generation sequencing, exome sequencing (ES), or genome sequencing (GS) of a cohort of ~ 6500 mostly non-syndromic IRD cases, we identified five probands (Family 1_II-3, Family 2_II-4, Family 3_II-1, Family 4_II-1, Family 5_II-1) with rare bi-allelic *SCLT1* variants (V1-8, MAF < 0.00001997)(see Fig. 1A and Table 1). No additional disease-causing variants were present in any of the currently known IRD genes[24] that were able to explain the clinical phenotype.

Table 1

**Clinical characteristics andSCLT1variants of the IRD patients of our study cohort.** AC, allele count; ACMG, American College of Medical Genetics; AF, allele f gain; AL, acceptor loss; BBS, Bardet-Biedl syndrome; DL, donor loss; EAS, East Asian; EOSRD, early-onset severe retinal dystrophy; F, female; ID, intellectual dis congenital amaurosis; LP, likely pathogenic; M, male; n.a., not available; n-FE, non-Finnish Europeans; RCD, rod-cone dystrophy; SLS, Senior-Løken syndrome; significance; *, breakpoint coordinates obtained based on GATK-SV pipeline. 15, Katagiri 2018; 18, Morisada 2020; 19, Sakakibara 2020; #, this study. For eac significant prediction scores have been reported. See Supplementary Tables 4 and 5 for a complete list of prediction scores.

| Family_ patient | Research_ID | Age at initial visit; Gender | Diagnosis | Ethnicity | Non-retinal features | Ocular symptoms | BCVA | *SCLT1* variants | GnomAD v4_ AF \| AC | ACMG | As ph |
|---|---|---|---|---|---|---|---|---|---|---|---|
| 1_II-3 | URDGH_005 | 43 \| F | EOSRD | South Korean | None | Poor vision since age 8–9, nystagmus | LP - OD LP + OS | **V1**: c.1631A > G; p.(Lys544Arg) | 0.00001997 \| 32 (30 EAS, 2 n-FE) | **LP** (PS3; PM2; PM3sup; PP1; PP3) | EO RC LC BB 19 |
| | | | | | | | | **V2**: ex3-10dup (chr4:128968796_ 129045332dup)*; p.? | n.a. | **LP** (PM1; PM2; PM3; PM4) | EO |
| 2_II-4 | URDGH_033 | 57 \| M | RCD | South Korean | None | Poor vision since age 55 | 20/25 OU | **V1** | | | |
| | | | | | | | | **V3**: c.290 + 2732A > G; p.? | n.a. | **VUS** (PS3; PM2; PP1) | RC |
| 3_II-1 | KA559 | 22 \| M | RCD | Japanese | ID, cleft palate | Nyctalopia and reduced visual acuity since age 13 | Age: 22 20/100 OD 20/80 OS | **V1** | | | |
| | | | | | | | | **V4**: c.439G > T; p.(Ala147Ser) | 0.00001752 \| 28 (EAS) | **VUS** (PM2) | RC |
| 4_II-1 | OGI163_447 | 25 \| F | RCD | White | None | Nyctalopia since early childhood, reduced side vision since age 16 | Age 30: 20/50 OD 20/60 OS | **V5**: c.671T > G; p.(Leu224Arg) | 0.000001885 \| 3 (n-FE) | **VUS** (PM2; PP1) | RC |
| | | | | | | | | **V6** c.1706G > A; p.(Ser569Asn) | 6.22E-07 \| 1 (EAS) | **VUS** (PM2; PP1) | RC |
| 5_II-1 | F4419_07910 | 8 \| F | EOSRD | White | None | Poor vision since infancy (15 months), nystagmus | Age 8: 20/500 OD 20/640 OS Age 12: CF OU | **V7**: c.778_780del; p.(Glu260del) | n.a. | **LP** (PS3; PM1; PM2; PM4; PP1) | EO |
| | | | | | | | | **V8**: c.1439 + 5G > T; p.? | 6.30E-07 \| 1 (n-FE) | **LP** (PS3; PM1; PM2; PP1) | EO |
| 5_II-2 | F4419_11487 | 5 \| M | EOSRD | White | None | Poor vision since infancy, nystagmus | Age 7: 20/640 OD 20/800 OS | **V7** | | | |
| | | | | | | | | **V8** | | | |

Of the eight likely causal variants found in *SCLT1*, four were missense (c.1631A > G p.(Lys544Arg); c.439G > T p.(Ala147Ser); c.671T > G p.(Leu224Arg); c.1706G > A p.(Ser569Asn)), one was a single amino acid deletion (c.778_780del p.(Glu260del)), two were non-coding variants c.290 + 2732A > G and c.1439 + 5G > T located in intron 5 and 16, respectively, and one was an exon 3–10 tandem duplication (hg38: chr4:128968796_129045332dup).

All variants were novel, except for c.1631A > G; p.(Lys544Arg) which was first reported in syndromic patients with SLS[15] or BBS[18,19] showing ocular phenotypes of EOSRD or RCD, respectively (See Supplementary Tables 1 and 2). The p.(Lys544Arg) is the second most commonly reported variant in *SCLT1* patients (See Supplementary Tables 1 and 2), and the most recurrent in our cohort: present heterozygously in three East Asian probands (families 1–3, see Fig. 1A). This allele is most likely a founder allele in the East Asian population, given its prevalence in this population (gnomAD v4.1: 30 alleles in total, MAF: 0.0006701), compared to the general population except for the East Asian (2 alleles, MAF: 0.00001997)[25].

Compound heterozygosity was confirmed by familial segregation analysis (families 1, 3, 4, and 5) and by using the gnomAD v2 Variant Co-Occurrence tool (https://gnomad.broadinstitute.org/variant-cooccurrence) (family 3) (see Supplementary Fig. 1). Unfortunately, we could not validate the phase of the variants identified in family 2, the p.(Lys544Arg) and the c.290 + 2732A > G, due to unavailability of parental samples and the absence of the c.290 + 2732A > G allele in gnomAD. In addition, the two variants were too far apart (~ 94 kb) to be cloned in one single fragment for phase determination. However, considering the allele count of both variants in gnomAD v4 (32 vs 0), and that p.(Lys544Arg) has been found in compound heterozygosity with various other *SCLT1* alleles in patients, we considered these two variants are likely in *trans*.

### Clinical phenotypes

Five probands (three females and two males) with *SCLT1*-associated disease had clinical diagnosis of EOSRD or RCD (see Fig. 2, Table 1, Supplementary Fig. 2 and Supplementary Table 3). Four probands manifested the onset of symptoms during infancy (~ 15 months), childhood or early teenagerhood (13 years), while family 2 proband reported a late-onset RCD phenotype at age 57 (see Table 1).

Visual acuity was variable across our clinical diagnosis. Family 1 proband, diagnosed with EOSRD and presented with light perception when evaluated at age 43, while family 2 and 4 RCD probands showed normal (20/25) or near normal (20/60) visual acuity in each eye at age 57 and 30, respectively.

Visual field data using Goldmann kinetic or Humphrey visual perimetry showed constriction of the visual fields in all probands, except for family 1 proband, for whom testing was not possible due to nystagmus (see Supplementary Table 3). Family 2 proband had ring scotoma around the center, while family 3 and 4 probands had constricted visual fields consistent with RCD. Full-field ERGs were available for all patients except for family 1 proband (see Supplementary Table 3). Family 2 proband showed varying degrees of scotopic alterations with less severe photopic dysfunction, while probands of family 3, 4, 5, and her brother had severe generalized dysfunction of both scotopic and photopic responses.

Fundus evaluation of family 2 proband was normal while in all other patients showed features that were typical for the retinal degeneration diagnosis (see Fig. 2 and Supplementary Fig. 2). Both siblings of family 5, affected by EOSRD, exhibit a large number of mottled and whitish lesions in the peripheral retina (see Supplementary Fig. 2). Spectralis OCT images were available for four individuals and showed significant alteration of the ellipsoid zone (EZ) bands and outer nuclear layer thinning, with small island of EZ bands at the foveal center (see Supplementary Table 3). Additional ophthalmic diagnoses included nystagmus in probands of family 1, 5, and her brother. Clinical examination of family 3 proband also revealed some degree of intellectual disability and cleft palate, but no other systemic diagnoses of note were present in our probands.

##### Four SCLT1 variants lead to splicing defects

Since three variants were located in proximity of canonical splice sites (V1 c.1631A > G p.(Lys544Arg), V7 c.778_780del p.(Glu260del), V8 c.1439 + 5G > T) and one variant was deep-intronic (V3 c.290 + 2732A > G), we tested their effects on pre-mRNA splicing with mini-gene constructs in HEK293T cells or by RT-PCR of blood-derived total RNA, when available (see Fig. 3 and Supplementary Fig. 3). Variants were predicted to affect normal splicing according to multiple in silico tools, such as SpliceAI[26], Pangolin[27], and ESEfinder[28] (see Supplementary Table 4).

The c.1631A > G p.(Lys544Arg) variant is located two base pairs (bp) upstream of the canonical donor splice site of exon 17 and is predicted to induce donor loss and activation of an alternative donor splice site located 6- bp upstream, thus resulting in the in-frame deletion of the last 6 nucleotides coding for two aminoacid residues (Val543 and Lys544) (see Supplementary Table 4). However, transcript analysis in families 1 and 2 probands, heterozygotes for this variant, only confirmed a fully penetrant donor loss inducing exon 17 skipping, but not the 6-bp deletion event (see Fig. 3A). This results validated the main molecular defect described by Katagiri and colleagues[15].

The deep-intronic variant c.290 + 2732A > G resulted in the partial inclusion of a 45-bp cryptic exon in intron 5, containing a stop codon. Although, the SpliceAI predictions were low (simultaneous acceptor and donor gain with scores of 0.16 and 0.15, respectively) (see Supplementary Table 4), the A > G transition was predicted by ESEfinder to enhance binding of three exonic splicing enhancers (ESEs) located within the cryptic exon (see Fig. 3B). The presence of the cryptic exon 5 was validated by RT-PCR of family 2 proband and control blood-derived RNA. Even though the cryptic exon inclusion was seen in both samples, the NGS quantification of cruptic exon reads including the reference allele (c.290 + 2732A, 32%) compared to the variante allele (c.290 + 2732G, 68%) revealed two-fold enrichment in the cryptic exon inclusion due to the c.290 + 2732A > G variant. Next-generation sequencing of the amplified product also confirmed the cryptic exon size of 45bp (see Fig. 3B).

The splicing effect of variants c.778_780del p.(Glu260del) and c.1439 + 5G > T was assessed via mini-gene based splicing assay. The first variant removes the first three bp of exon 11, leading to exon 11 skipping, with a smaller fraction of the transcripts still containing the exon but lacking the Glu260 (see Fig. 3C). The c.1439 + 5G > T variant, located in intron 16, showed incomplete missplicing effect with partial skipping of exon 16, consistent with the Pangolin prediction (splice loss 0.65). The same donor loss was not predicted by SpliceAI (0.12), which instead predicted a donor gain 80-bp upstream (0.33) of the variant, not confirmed by the splicing assay (see Fig. 3D and Supplementary Table 4).

Due to the partial effect on cryptic exon inclusion, we consider the c.290 + 2732A > G as a hypomorphic variant and was classified as variant of uncertain significance (VUS) according to the ACMG guidelines, while the other three variants were classified as likely pathogenic (see Table 1).

##### Detection of a large structural variant in SCLT1

Structural variants (SVs) analysis of the GS data in the proband of family 1 by GATK-SV pipeline led to the identification of a novel ~ 76kb duplication (chr4: 128,968,796 – 129,045,332) encompassing the genomic sequence between exon 3 and 10 of *SCLT1* and never reported in gnomAD SVs v4.1 (see Figs. 1 and 4A).

This SV is supported by B-allele frequency, paired-end, read-depth, split reads on both SV sides (see Fig. 4A). Although the lack of additional patient DNA did not allow us to Sanger validate the breakpoint coordinates provided by the GATK-SV pipeline, we were able to demonstrate the exon 3–10 in frame duplication by RT-PCR using patient blood-derived RNA (see Fig. 4B). Using primers spanning the predicted duplication boundary, we amplified a 142-bp fragment corresponding to the exon 10 - exon 3 splicing boundary (see Fig. 4B). This fragment was specific to the patient RNA and was not detected using control human RNA. The coding effect of this novel SV consists in the incorporation of 225 amino acids (Lys35 - Lys259) between residue Lys259 and Glu260, which are part of the Myosin Tail Domain (MTD). In the absence of a SCLT1 protein structure model on Protein Data Bank, we used the Alphafold Server3 (https://alphafoldserver.com) to evaluate the structural changes caused by the in-frame exon 3–10 duplication (see Supplementary Fig. 4). Increased intra-protein interactions were predicted following the SCLT1 exon 3–10 duplication. Furthermore, the wild-type elongated linker protein is predicted to become shortened due to the duplication, potentially leading to a loss of function.

### Protein modelling and genotype-phenotype correlation analysis

Variants in *SCLT1* have been associated with syndromic forms of retinal degeneration. To investigate whether this phenotypic variability was the consequence of a specific variant localization, we plotted the known 11 *SCLT1* variants reported in the literature and the seven novel variants detected in our probands onto the secondary structure of the human SCLT1, a 688 amino acid protein (UniProtKB ID: Q96NL6) (see Fig. 1B and Supplementary Table 1)[12–23]. A third of the 18 analyzed *SCLT1* variants were missense while the others were either truncating or non-coding variants. Variants were equally distributed within the secondary structure of the protein, affecting either its disorganized N-terminal region (residues 1-250) and the three functional domains at the central (MTD) and C-terminal (V-pump and CTD) region (see Fig. 1B). Of note, all variants associated with syndromic phenotypes were splice variants affecting the donor splice site, located either at the terminal of an exon (c.1218G > A; p.Met406Ile and c.1631A > G; p.Lys544Arg) or at conserved intronic positions + 1, +2, and + 3, suggesting a severe truncating effect. Among the variants associated with non-syndromic IRDs there were five missense changes and splicing variants with incomplete effect such as the c.290 + 2732A > G and the c.1439 + 5G > T (see Fig. 3). The mutation tolerance at SCLT1 protein residues was analyzed using MetaDome (https://stuart.radboudumc.nl/metadome/)[29], while the impact of specific missense variants on SCLT1 structure and function was predicted by tools like SIFT[30], PolyPhen2[31], CADD Phred[32], and REVEL[33] (see Supplementary Table 5). The five missense variants (p.Ala147Ser, p.Leu224Arg, p.Arg276His, p.Lys544Arg, p.Ser569Arg) present in non-syndromic cases, four of which identified in our patients, were all predicted deleterious by CADD Phred, and by at least one between SIFT or PolyPhen2 prediction programs, but not by Revel (see Supplementary Table 5). Moreover, three of them (p.Ala147Ser, p.Leu224Arg, p.Arg276His), were also located in residues predicted to be tolerant toward mutations (see Fig. 1B). The p.(Lys544Arg), enriched among the East Asian population, was the only variant shared between non-syndromic and syndromic cases. In particular, it was detected heterozygously in two unrelated Japanese families with BBS plus RCD[18] and in a third Japanese family with SLS plus EOSRD[15] (see Fig. 1B and Supplementary Table 1).

Thus, after comparing genotypes of alle previously reported and new 23 cases carrying bi-allelic *SCLT1* variants (see Supplementary Table 1), we observed an enrichment of hypomorphic alleles in non-syndromic IRD patients compared to syndromic *SCLT1* patients.

## DISCUSSION

In this retrospective study, we describe five probands with clinical phenotypes of retinal degeneration (EOSRD or RCD) associated with rare bi-allelic variants in *SCLT1*, a gene first associated with recessive ciliopathies OFD-IX, BBS, and SLS. We identified seven novel variants, three of which were missense (p.Ala147Ser, p.Leu224Arg, p.Ser569Asn), four affecting splicing (c.290 + 2732A > G, p.Glu260del, c.1439 + 5G > T, p.Lys544Arg), and a ~ 76kb in-frame tandem duplication (chr4:128,968,796_129,045,332dup) encompassing exon 3–10 of the gene. Our study thus further supports the association of *SCLT1* variants with non-syndromic IRDs first described by de Castro-Miró and colleagues[22] and expands the number of non-syndromic cases.

Both the deep-intronic c.290 + 2732A > G and the tandem duplication are, to our knowledge, the first variants of their class to be described in *SCLT1* (see Supplementary Table 1). However, although for the first one it was possible to experimentally validate its pathogenicity, that was not possible for the structural variant. The in-frame tandem duplication is not predicted to disrupt any known protein domains, yet it is predicted to double the N-terminal region, extending the protein by 30% of its original length and increasing the number of intra-protein interactions. Therefore, misfolding followed by subsequent protein degradation are a likely molecular consequence for this structural variant.

By comparing the variants identified in non-syndromic *vs* syndromic patients and their location on the secondary structure of SCLT1 protein, we did not identify a clear mutation hotspot able to explain the different clinical phenotypes. However, by looking at all the bi-allelic combinations in currently reported syndromic cases, we noted a clear enrichment for bi-allelic null alleles, and hypothesize a model in which at least one hypomorphic allele may alleviate the syndromic phenotype and lead to an isolated retinal degeneration (see Fig. 1B). However, with only 28 *SCLT1* patients described thus far, we cannot draw final conclusions, and additional cases will be needed to support our observation. Equally, we cannot rule out the presence of genetic modifiers of disese severity[35].

With the exception of the family 2 proband, a notable feature in our study cohort was the early disease onset, with symptoms beginning prior to the age of 13 and as early as 15 months, in the siblings of family 5. In the latter family, the large number of mottled and whitish lesions in the peripheral retina resembled those described in the fundoscopies of LCA patients harboring bi-allelic variants in *CEP290*, another ciliopathy gene[36]. The early-onset retinal disease observed in our cases overlaps with the previous ophthalmic diagnoses of *SCLT1* patients.

None of the probands in our cohort complained of any syndromic symptoms, except for family 3 proband, in whom mild intellectual disability and cleft palate were noted. Although these extra ocular phenotypes overlap with OFD-IX features, they are milder than reported for this syndrome and they do not include all of the OFD-IX symptoms[7,8].

The most recurrent allele in our study cohort was the known variant c.1631A > G (p.Lys544Arg), found in two South Korean and one Japanese family (Family 1–3). This variant is currently the second most recurrent *SCLT1* allele in patients (second only to c.290 + 2T > C, see Supplementary Tables 1 and 2), yet it is undoubtedly the most frequent in the general population based on gnomAD v4.1 data (AF = 0.00001997 *vs* AF = 6.586e-7 of c.290 + 2T > C). That is likely due to its enrichment among the East Asian population, especially in Japan where this allele has an allele frequency of 0.1%[37].

Using cDNA from two different probands carrying the c.1631A > G (p.Lys544Arg) variant we have independently validated finding by Katagiri and colleagues[15], that the variant leads to a partial exon 17 skipping (see Fig. 3A). Since functional characterization of this variant was performed on blood-derived RNA, we cannot be sure which of the effects is predominant in human photoreceptors: exon skipping or the p.Lys544Arg missense. Both events will affect the C-terminal domain, an area that is predicted to poorly tolerate change, and is known (amino acids 541–688) to contain dileucine motifs that may bind clathrin[38] and AP2 complex[39]. Therefore, both exon 17 deletion and the missense are likely to be pathogenic.

Of the seven novel variants reported in our study, only three were likely pathogenic, while three missense (p.Ala147Ser, p.Leu224Arg, p.Ser569Asn) and the deep-intronic variant c.290 + 2732A > G were classified as VUS. The p.(Leu224Arg) and p.(Ser569Asn) were found in the family 4 proband and her brother, and were proven to be in *trans*. The proband of this family presented with an early-onset disease. The p.(Ser569Asn) is located in the CTD domain and predicted to be in a less mutation tolerant region, according to MetaDome (see Fig. 1B). Regarding the p.(Ala147Ser) and the c.290 + 2732A > G, we could hypothesize a milder effect for both alleles, as they were detected in patients with the age of onset of 13 and 55 years, respectively. The p.(Ala147Ser) was predicted to modify a tolerant region and transcript analysis of c.290 + 2732A > G showed a moderate effect, suggesting that both variants could act as hypomorphic alleles. Both alleles were found in *trans* with the severe variant p.(Lys544Arg), indicating that a likely hypomorphic second allele could induce a milder retinal disease phenotype. Moreover, it is important to mention that under the experimental settings of our mini-gene splicing assay, the strength of the observed splicing effect is approximate and we cannot rule out that the c.290 + 2732A > G might have a more severe molecular effect when tested in a larger genomic context and in the retina.

In conclusion, our data validate the phenotypic expansion caused by pathogenic variants in *SCLT1* and expand the mutation landscape of this gene by providing novel coding and non-coding variants in this ciliopathy gene.

### METHODS

### Ethics statement

The study was approved by the institutional review board of all participating institutions (Institutional Review Board of Gangnam Severance Hospital: 3-2021-0153 for families 1 and 2, NHO Tokyo Medical Center (Reference; R19-030, R 21–108, R22-028) for family 3, Partners HealthCare System for family 4, and Committees of Protection of Persons Ile de France V for family 5) and adhered to the Declaration of Helsinki. Informed consent was obtained from all individuals on whom genetic testing and further molecular evaluations were performed.

### Clinical evaluation

Five probands with autosomal recessive rod-cone degeneration were enrolled in this study. Two of them were ascertained from Gangnam Severance Hospital, while the other three from NHO Tokyo Medical Center, Massachusetts Eye and Ear, and the National Reference Centre of Rare Diseases at Quinze-Vingts National Hospital.

Clinical evaluation was performed by experienced ophthalmologists according to previously published protocols and included functional and structural assessments[40–43].

#### Genetic analysis

Blood samples were obtained from probands, and when possible, their parents. DNA was isolated from peripheral blood lymphocytes by standard procedures. GS for proband of family 1 and 2 was performed at Theragen Bio (Seongnam-si, South Korea). GS data was aligned to hg38, and variant calling was performed using DRAGEN-GATK4 best practice. GATK-structural variant (SV) cohort mode using 5 different algorithms was used for structural variant calling[44]. Family 4 proband is part of a historical cohort that underwent clinical evaluation in the Inherited Retinal Disorder Service (at MEE; Boston, MA) in the 1990s and early 2000s. Exome sequencing (ES) for this proband was performed at the Center for Mendelian Genomics at the Broad Institute of the Massachusetts Institute of Technology and Harvard using methodology described previously[45]. ES data were aligned to hg38 and variants were called using the GATK HaplotypeCaller package version 3.5 (https://software.broadinstitute.org/gatk/). Data were displayed and analyzed with an online tool (https://seqr.broadinstitute.org)[46].

Family 5 proband was screened applying a customized NGS panel as reported before[47] updated regularly to include newly IRD-associated genes, while NGS-based testing was performed by commercial diagnostic laboratories (Blueprint Genetics) for the proband in family 3.

To detect possible copy number variations, gCNV software was used as before[48]. Relatedness of the sequenced families was excluded using Peddy[49].

#### Variant validation and phasing

All presented variants refer to the *SCLT1* transcript NM_144643.4. Variant segregation was performed by Sanger sequencing (see Supplementary Table 6 for sequencing primer) or analysis of NGS reads. In family 2 proband, the lack of genomic DNA sample from other family members and the significant distance between the two *SCLT1* variants detected in this proband did not allow us to determine phase experimentally. Also, since one of variant (c.290 + 2732A > G)

was absent from gnomAD database, we could not validate phase using the Variant Co-Occurrence tool (https://gnomad.broadinstitute.org/variant-cooccurrence). However, given that the second *SCLT1* variant (p.Lys544Arg) had a higher allele count of 32 instances on gnomAD, we could consider these two variants likely to be in *trans*. Sanger sequencing was performed on ABI 3730xl (Applied Biosystems) using BigDye Terminator v3.1 kits (Life Technologies). Sequence analysis was done using SeqManPro (Lasergene 11, DNAStar Madison, WI, USA).

## Mini-gene splicing assay

A mini-gene splicing assay was performed using a mini-gene split GFP construct[50], in which N and C-terminal parts of the GFP gene were separated by SMN1 introns 7 and 8 (NM_000344). Reference and mutated gene fragments containing 400-500bp including and surrounding intron 5, exons 11, 16, or 17 of *SCLT1* gene flanked with 30bp vector homology arms were synthetized (TWIST Bioscience, USA) and cloned into the mini-gene construct (Gibson Assembly Master Mix, New England Biolabs). After Sanger sequencing verification of all constructs, they were transfected into the HEK293 cells (Lipofectamine 3000, Thermo Fisher Scientific). Forty-eight hours post transfection, total RNA was extracted from the transfected cells (RNAeasy Mini Kit, Qiagen) and cDNA was generated using random hexamer primers (SuperScript IV Synthesis Kit, Thermo Fisher Scientific). Subsequently, the mini-gene transcripts were amplified from the cDNA using primers specific to the split GFP fragments (F: 5’- CACACTGGTGACAACATTTACATAC-3’; R: 5’- GAAATCGTGCTGTTTCATGTGATC-3’). The PCR products were column purified (DNA Clean & Concentrator-5, Zymo Research) and analyzed by Sanger sequencing.

**Protein modelling, prediction of missense variants, and variant classification.**

The mutation tolerance at *SCLT1* protein residues was analyzed using MetaDome (https://stuart.radboudumc.nl/metadome/)[29], while the impact of specific missense variants on *SCLT1* structure and function, was predicted by using five prediction algorithms: SIFT[30], PolyPhen2[31], CADD Phred[32] REVEL[33], and EVE[34]. Variants were finally classified according to the ACMG guidelines[51]. Protein modelling of SCLT1 was done with Alphafold3 and UCSF Chimera X[52,53].

## Declarations

#### DATA AVAILABILITY

Variants are available through dbGAP (phs001272.v1.p1 and phs002459.v1.p1) and ClinVar (submission number SUB15213167).

#### CODE AVAILABILITY

Software used in this study: Biorender; SeqManPro (Lasergene v11); Seqr (https://seqr.broadinstitute.org/); UCSF Chimera X.

#### ACKNOWLEDGEMENTS

This work was supported by grants from the National Eye Institute [R01EY035717 (KMB), and P30EY014104 (MEEI core support)], the Foundation Fighting Blindness (EGI-GE-1218-0753-UCSD, KMB), Iraty Award 2023 (KMB), Lions Foundation (KMB), Research to Prevent Blindness Unrestricted Grant (KMB) and the Curing Kids Foundation (KMB). Exome sequencing and analysis were provided by the Broad Institute of MIT and Harvard Center for Mendelian Genomics (Broad CMG) and was funded by the National Human Genome Research Institute, the National Eye Institute, and the National Heart, Lung and Blood Institute grant UM1HG008900 and in part by National Human Genome Research Institute grant R01 HG009141.

The authors would like to thank the patients and their family members for their participation in this study and the Ocular Genomics Institute Genomics Core members for their experimental assistance. The authors would like to thank the Exome Aggregation Consortium, the Genome Aggregation Database (gnomAD) and the groups that provided exome variant data for comparison. A full list of contributing groups can be found at http://exac.broadinstitute.org/about and http://gnomad.broadinstitute.org/about. We thank Prof. Dr. Fowzan S. Alkuraya and Lama AlAbdi, PhD, from the Department of Translational Genomics, Center for Genomic Medicine, King Faisal Specialist Hospital and Research Center, Riyadh, Saudi Arabia; Prof. Dr. Naoya Morisada from the Department of Pediatrics, Kobe University Graduate School of Medicine, Kobe, Japan; Dr. Lev Prasov from the Department of Ophthalmology and Visual Sciences, Kellogg Eye Center, University of Michigan, Ann Arbor, Michigan; Prof. Dr. Katarina Stingl and Dr. Susanne Kohl from the University Eye Hospital and the Institute for Ophthalmic Research, Center for Ophthalmology, University of Tübingen, Tübingen, Germany, for their help with the clinical interpretation of some *SCLT1* variants.

DNA samples from the French center originate from NeuroSensCol DNA bank (PI: JA Sahel, coPI I Audo, partner with Centre Hospitalier National d’Ophtalmologie des Quinze-Vingts, INSERM and CNRS). Funding from this group came from LABEX LIFESENSES (ANR-10-LABX-65) supported by French state funds managed by the Agence Nationale de la Recherche within the Investissements d’Avenir program (ANR-11-IDEX-0004-0) and IHU FOReSIGHT (ANR-18-IAHU-0001), and from the National Reference Centers for Rare Diseases Program.

Funding from South Korea group came from Korea Disease Control and Prevention Agency (grant No. 2018-ER6902-02) and the National Research Foundation of Korea grant funded by the Korean government (MIST) (grant No. 2020R1C1C1007965).

#### AUTHOR CONTRIBUTIONS

R.S. performed most of the experiments, data analysis, and led the manuscript writing. K.F., E.M.P., C.Z., I.A., analyzed clinical data and reviewed the manuscript. S.H.B., J.N., C.C., S.D.T., performed part of the genetic analysis. K.M.B. and J.H. guided the experimental design, aided in variant analysis, and contributed to writing the manuscript.

#### COMPETING INTERESTS

The authors declare no conflicts of interest.

### Figures

## Figure 1

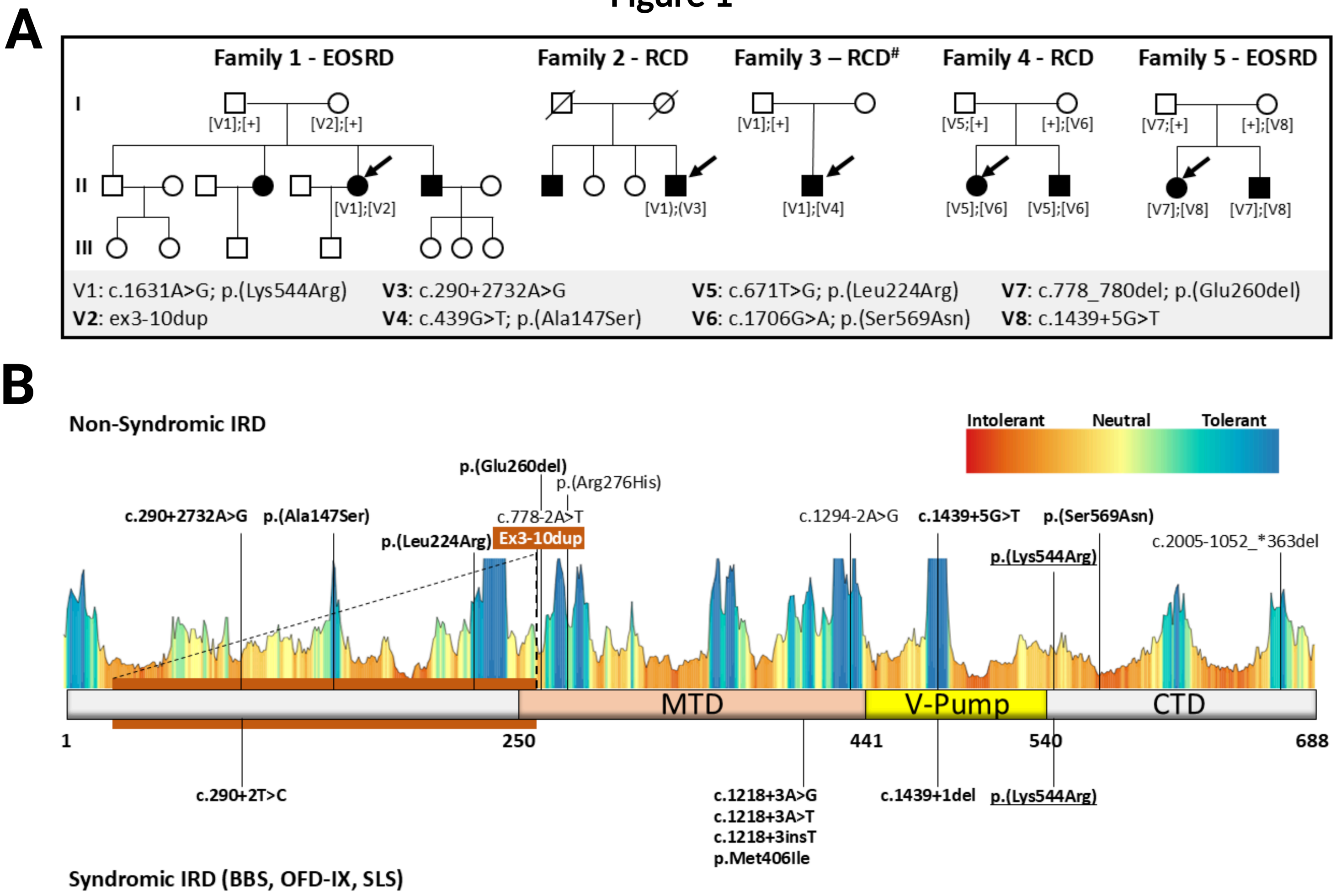

Figure 1

**Pedigrees of the *SCLT1* families described in this study. (a)** For each family (1-5), the specific IRD phenotype diagnosed is mentioned above each pedigree (EOSRD, early-onset severe retinal dystrophy; RCD, rod-cone dystrophy). Family 3, in which a mildly syndromic proband was identified, is indicated with a hashtag (#). Affected male and female subjects are represented with black squares or circles, respectively. Probands are indicated by a black arrow. Novel variants (V2-V8) are indicated in bold. All presented variants refer to the *SCLT1* transcript NM_144643.4. Bi-allelic inheritance was confirmed by familial segregation analysis (families 1,4 and 5), or by using the gnomAD v2 Variant Co-Occurrence tool (family 2 and 3). **(b)** SCLT1 secondary structure and distribution of known and novel disease variants found in affected individuals. Prediction model of the mutation tolerance landscape of the SCLT1 protein was retrieved from MetaDome webpage. Protein motifs and catalytic domain are highlighted using different colors, while variants were divided in two groups, depending whether they were found in syndromic or non-syndromic IRD patients. Known variants were retrieved from the Leiden Open Variation Database (LOVD). Novel variants reported in this study are in bold while the known p.(Lys544Arg), present in both patient groups, is highlighted. OFD-IX, orofaciodigital syndrome type-IX; BBS, Bardet-Biedl syndrome; SLS, Senior-Løken syndrome; MTD, myosin tail domain; CTD, C-terminal domain.

## Figure 2

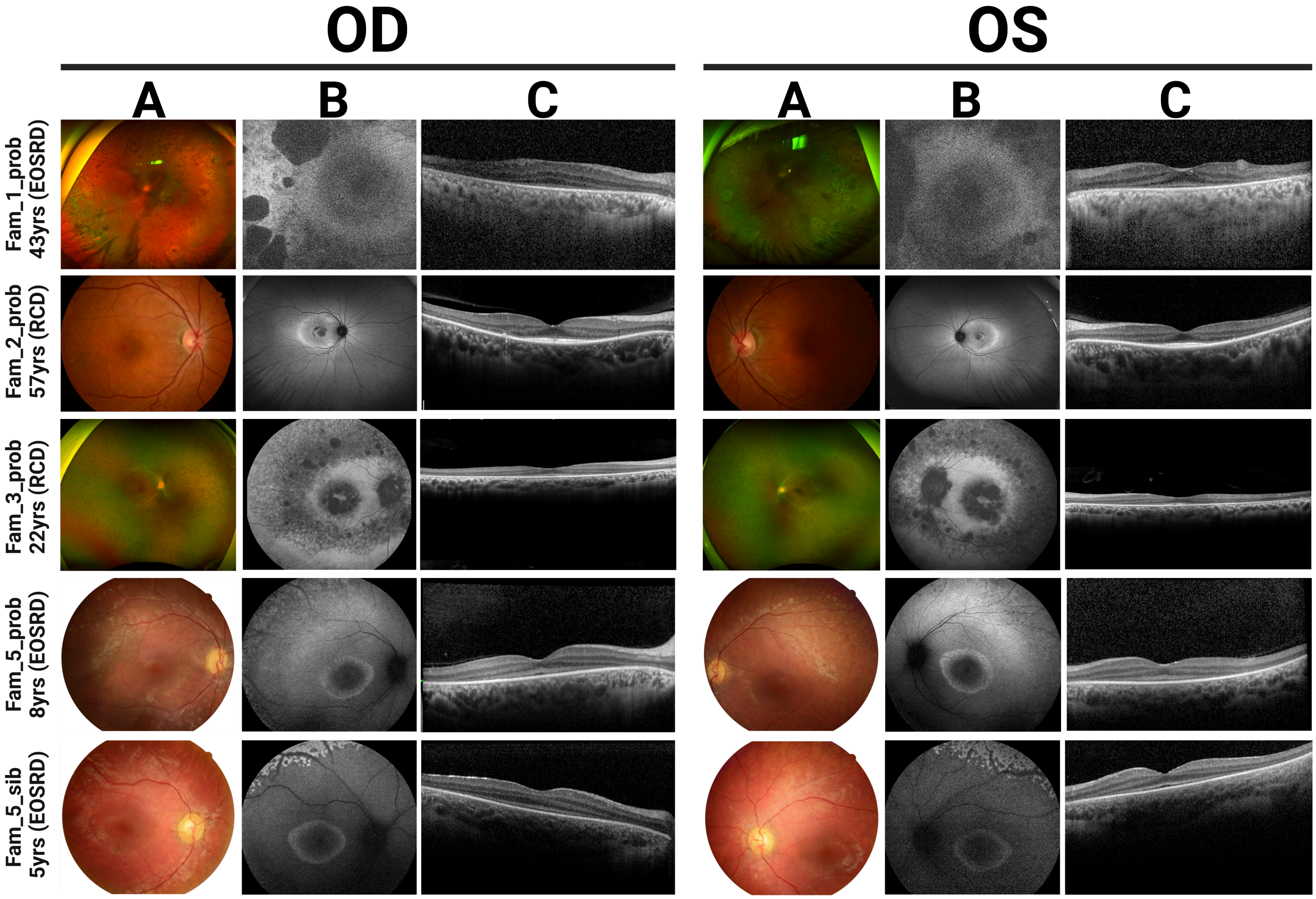

**Figure 2**

**Retinal phenotypes of *SCLT1*-IRD patients.** Images show fundus photos (A), fundus autofluorescence (B), and OCT imaging (C) for four probands and the affected sibling of family 5 proband. The age (in years) indicate when the imaging data were obtained, which not always overlaps with the actual age of onset. Imaging showed features consistent with the clinical diagnosis. The specific IRD phenotype of each patient is given in brackets (EOSRD, early-onset severe retinal dystrophy; RCD, rod-cone dystrophy).

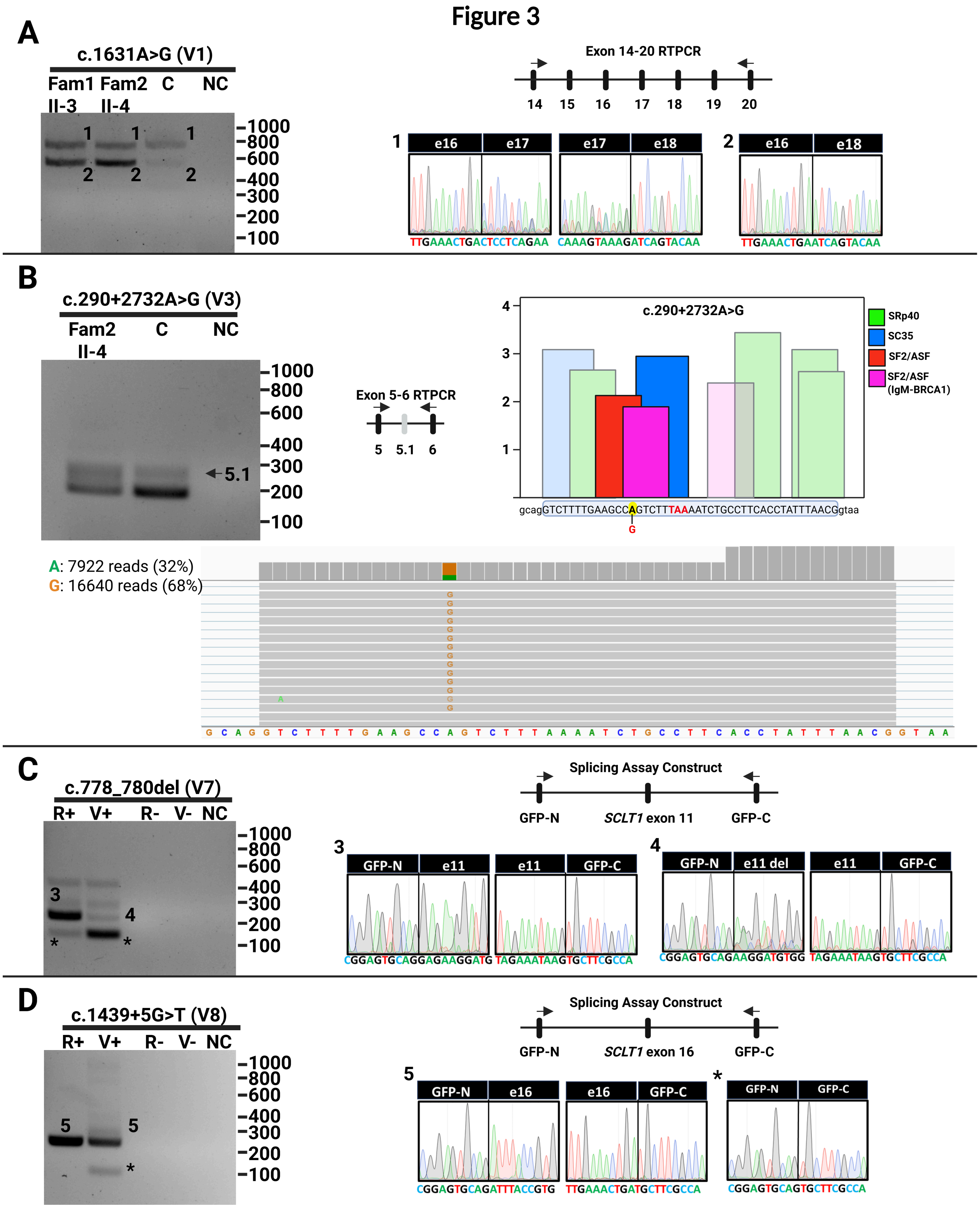

Figure 3

**Functional validation of *SCLT1* splicing variants.** **(a)** Transcript analysis of variant c.1631A>G; p.(Lys544Arg) using primers spanning *SCLT1*exon 14 and 20. RT-PCR revealed exon 17 skipping in probands of family 1 and 2, heterozygotes for the variant, compared to the reference control (C) and negative control (NC). RT-PCR bands were purified and sequenced, confirming the splicing defects. **(b)** Transcript analysis of variant c.290+2732A>G using primers spanning *SCLT1* exon 5 and 6. RT-PCR revealed cryptic exon 5.1 inclusion skipping in probands II-4 of family 2, heterozygotes for the variant. The transcript defect was also detected in the RNA of the healthy control (C) who did not carry the variant, although its intensity was reduced. ESEfinder analysis of the 45-bp cryptic exon activated by the deep-intronic variant c.290+2732A>G compared to the reference sequence. The A>G transition does not occur at the splicing boundaries and leads to the activation of SC35 and SF2/ASF exonic splicing enhancers (ESEs). Next-generation sequencing was used to quantify the percentage of reads showing cryptic exon inclusion, which accounted for 68%. **(c)** Reference (R) and mutant (V) splicing constructs for variants c.778_780del; p.(Glu260del), and c.1439+5G>T **(d)** were designed, transfected in HEK293-T cells and assayed for splicing defects. Transcript analysis by RT-

PCR (+ and – refer to RT+ and RT- ) using primers spanning the flanking GFP exons unraveled pre-mRNA defects for both *SCLT1*variants. RT-PCR bands were purified and sequenced, confirming the splicing defects.

### Figure 4

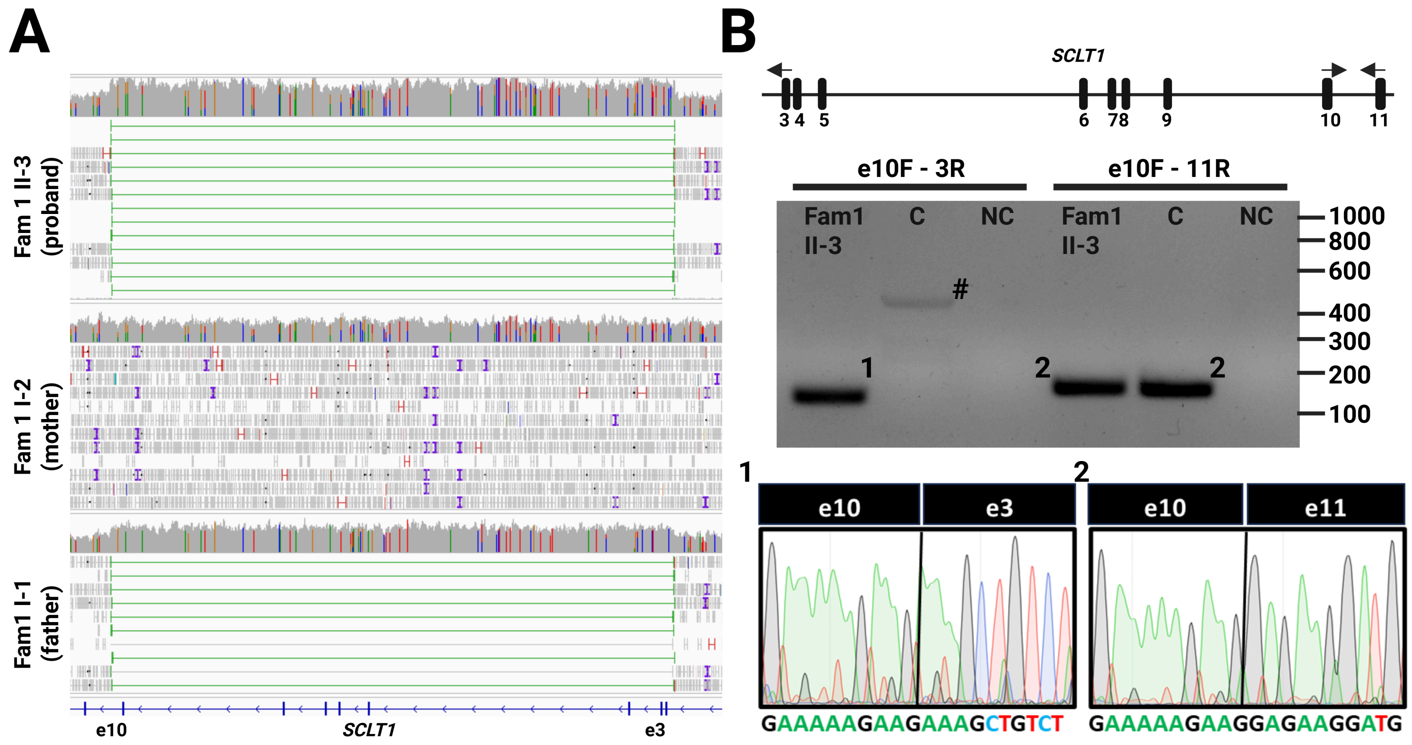

Figure 4

***SCLT1* exon3-10 in frame tandem duplication detected in proband I-3 (a)** IGV tracks showing the segregation of the tandem duplication in Family 1. Indeed, the structural variant is detected in the proband and her father (healthy carrier) but not in her mother. **(b)** RT-PCR-based validation of the duplication breakpoint in proband II-3 using ad hoc primers spanning exon 3 and exon 10. The resulting ~150-bp amplicon could be detected in the proband, but it is absent when using a control (C) sample and negative control (NC). A control RT-PCR amplicon encompassing exon 10 and 11 showed no difference between proband and control sample.

### Supplementary Files

This is a list of supplementary files associated with this preprint. Click to download.

- SupplementaryTablesApril22.xlsx
- SupplementaryFiguresApril22.pdf
